# 伴低T3综合征的急性髓系白血病患者的临床特征和预后分析

**DOI:** 10.3760/cma.j.issn.0253-2727.2021.11.007

**Published:** 2021-11

**Authors:** 兰竹 李, 金花 胡, 子瑶 徐, 鸣 洪, 倩 孙, 思轩 钱, 文洁 刘

**Affiliations:** 南京医科大学第一附属医院、江苏省人民医院血液科 210029 Department of Hematology, the First Affiliated Hospital of Nanjing Medical University, Jiangsu Province Hospital, Nanjing 210029, China

**Keywords:** 低T3综合征, 白血病，髓系，急性, 临床状态, 预后, Low T3 syndrome, Leukemia, myeloid, acute, Clinical status, Prognosis

## Abstract

**目的:**

探讨伴低T3综合征（LT3S）的急性髓系白血病（AML）患者的临床特征及预后。

**方法:**

回顾性分析2013年1月至2019年12月江苏省人民医院血液科连续收治的236例AML患者的临床资料，按照血清甲状腺素水平将其分为LT3S组和非LT3S组，比较两组患者的临床特征及预后。

**结果:**

在236例AML患者中，有62例（26.3％）患者出现LT3S。血清游离三碘甲状腺原氨酸（T3）水平与白蛋白（*r*＝0.443，*P*<0.001）、血红蛋白（*r*＝0.187，*P*＝0.005）水平呈正相关，与C反应蛋白（*r*＝−0.406，*P*<0.001）、乳酸脱氢酶（*r*＝−0.274, *P*<0.001）水平呈负相关。LT3S组与非LT3S组相比，总生存（OS）期（7.5个月对29.9个月，*P*<0.001）和无进展生存（PFS）期（2.0个月对24.0个月，*P*<0.001）明显缩短。使用倾向性匹配评分均衡患者基线资料后显示，LT3S组与非LT3S组相比OS期（9.6个月对30.4个月，*P*＝0.010）和PFS期（3.0个月对30.0个月，*P*＝0.014）仍明显缩短。合并LT3S是影响AML患者OS（*HR*＝2.553，95％ *CI* 1.666～3.912，*P*<0.001）和PFS（*HR*＝1.701，95％ *CI* 1.114～2.597，*P*＝0.014）的独立危险因素。亚组分析提示，在肥胖、体能状态差或采用标准方案化疗的AML亚组中合并LT3S者预后更差。

**结论:**

LT3S的发生反映AML患者临床状态差，不能耐受高强度化疗，预后不良。

## Abstract (en)

**Objective:**

To investigate the clinical features and prognosis of low triiodothyronine syndrome (LT3S) in patients with acute myeloid leukemia (AML).

**Methods:**

A total of two 236 patients with AML who presented at the Jiangsu Provincial Hospital between January 2013 and December 2019 were included, and their data were retrospectively reviewed. The patients were divided into two groups, including the LT3S group and the non-LT3S group, according to their serum thyroxine level. The clinical characteristics and prognosis of the two groups were compared.

**Results:**

Among the 236 patients, 62 (26.3％) patients had LT3S. Serum-free T3 level was positively correlated with albumin (*r*＝0.443, *P*<0.001) and hemoglobin (*r*＝0.187, *P*＝0.005) levels and negatively correlated with C-reactive protein (*r*＝−0.406, *P*<0.001) and lactate dehydrogenase (*r*＝−0.274, *P*<0.001) levels. The overall survival (OS) (7.5 months *vs* 29.9 months, *P*<0.001) and progression-free survival (PFS) (2.0 months *vs* 24.0 months, *P*<0.001) were significantly shortened in the LT3S group compared with the non-LT3S group. After propensity score matching, the OS (9.6 months *vs* 30.4 months, *P*＝0.010) and PFS (3.0 months *vs* 30.0 months, *P*＝0.014) were still significantly reduced in the LT3S group compared with the non-LT3S group. Therefore, LT3S was an independent risk factor for OS (*HR*＝2.553, 95％ *CI* 1.666–3.912, *P*<0.001) and PFS (*HR*＝1.701, 95％ *CI* 1.114–2.597, *P*＝0.014) in patients with AML. Subgroup analysis suggested that patients with LT3S had a worse prognosis in patients with AML who were obese, fragile, or treated with standard chemotherapy.

**Conclusion:**

The occurrence of LT3S reflects the poor clinical status and prognosis of patients with AML.

急性髓系白血病（AML）是一种具有高度侵袭性的恶性肿瘤，异质性大，因此在治疗前进行准确的危险度分层对于选择合理的治疗方案尤为重要。细胞遗传学及分子生物学特征是AML预后分层的主要依据[1]。然而，患者的体能状态在治疗方案的选择和临床预后中同样占据重要地位。目前，我们主要通过美国东部肿瘤协作组（ECOG）评分、综合老年学评估（CGA）等量表对患者的体能状态进行评价，而缺乏相对客观的检验指标，因此我们希望寻找可靠、易于操作的检验指标用于AML患者个体风险评估。

低T3综合征（Low triiodothyronine syndrome，LT3S），也称为非甲状腺疾病综合征或正常甲状腺病态综合征[2]，是指在多种应激状态下出现的非甲状腺疾病引起的甲状腺激素代谢紊乱。临床上以血清总三碘甲状腺原氨酸（T3）和游离T3（FT3）水平降低，反T3增高，血清甲状腺素（T4）和促甲状腺激素（TSH）水平正常为特征。研究发现，在重症疾病状态下，约44％的患者伴有甲状腺激素水平异常，其中以LT3S最为常见，且其与患者的预后及远期生存率密切相关[3]。在多种疾病如心肌梗死和心衰[4]、终末期肾病[5]、肺癌[6]、乳腺癌[7]中LT3S是预后不良的指标。然而，目前尚无关于LT3S在AML患者中临床意义的研究。因此，本研究旨在探讨AML患者合并LT3S的临床特征，以及LT3S在AML患者中的预后意义。

## 病例与方法

1. 病例：本研究共纳入江苏省人民医院2013年1月至2019年12月连续收治的新诊断AML（不包括急性早幼粒细胞白血病）患者236例。所有患者的诊断均符合AML诊断标准[8]。其中，采用DCAG方案（地西他滨、阿糖胞苷、阿柔比星、G-CSF）治疗113例（47.8％），采用IA方案（去甲氧柔红霉素、阿糖胞苷）治疗73例（30.9％），采用DA方案（柔红霉素、阿糖胞苷）治疗27例（11.4％），采用HA方案（高三尖杉酯碱、阿糖胞苷）治疗14例（5.9％），采用姑息治疗9例（3.8％）。

2. 数据收集：基线临床特征包括年龄、性别、体重指数（BMI）、FAB分类、ECOG体能状态评分。收集患者治疗前WBC、HGB、PLT、乳酸脱氢酶（LDH）、白蛋白（ALB）、C反应蛋白（CRP）、NPM1突变、FLT3突变、TP53突变及骨髓原始细胞百分比等实验室数据。甲状腺功能检测采用化学发光免疫分析法（安图生物工程股份有限公司产品）。正常参考区间为：TSH 0.270～4.200 mIU/L，FT3 3.10～6.80 pmol/L，血清游离T4（FT4）12.00～22.00 pmol/L。LT3S定义为血清FT3浓度降低、FT4和TSH浓度在正常范围内或轻度降低。

3. 随访：通过电话及查阅门诊病历进行随访，末次随访时间为2020年6月30日，中位随访时间为37.8（23.3～50.3）个月。早期死亡定义为诊断后3个月内的死亡。总生存（OS）时间定义为自诊断至末次随访或因任何原因死亡的时间。无进展生存（PFS）时间定义为从完全缓解之日起至白血病复发或因任何原因死亡的时间。

4. 统计学处理：采用SPSS软件25.0版、R语言3.6.3版进行统计学分析，双侧*P*值<0.05为差异有统计学意义。组间分类变量的比较采用卡方检验或Fisher精确概率检验。采用 1∶1 最近邻匹配法，设定容差值为 0.1，将患者性别、年龄、治疗方案等基线资料作为处理变量，是否合并LT3S作为观察变量纳入匹配模型，进行倾向性匹配分析。倾向性匹配分析前后，分别采用 Kaplan-Meier 法绘制生存曲线，Log-rank 法比较生存曲线差异。采用Cox比例风险回归模型进行单因素和多因素分析，单因素分析中 *P*<0.1的变量纳入多因素分析。为保证结果的稳定性，我们还进行敏感性分析，分别将年龄>75岁、ECOG评分>3分、采用姑息治疗的患者排除，再次进行生存分析。此外，我们通过亚组分析来评估LT3S在不同亚组患者中的预后意义。

## 结果

一、一般特征

在236例AML患者中，男125例（53.0％），女111例（47.0％），中位年龄为61（47～69）岁。其中合并LT3S者62例（26.3％）。LT3S组血清FT3、FT4、TSH浓度分别为（2.30±0.57）pmol/L、（14.70±2.86）pmol/L、（1.57±1.04）mIU/L。非LT3S组FT3、FT4、TSH浓度分别为（4.30±0.72) pmol/L、（15.98±2.67) pmol/L、（2.08±1.00）mIU/L。此外，LT3S组中有19例（30.6％）患者FT3、FT4水平同步下降。我们分析了血清FT3和蛋白质代谢、贫血、炎症状态和肿瘤负荷之间的关系，结果显示FT3与ALB（*r*＝0.443，*P*<0.001）、HGB（*r*＝0.187，*P*＝0.005）呈正相关，与CRP（*r*＝−0.406，*P*<0.001）、LDH（*r*＝−0.274，*P*<0.001）呈负相关。

为了均衡LT3S组与非LT3S组患者之间的基线特征，我们将患者性别、年龄、治疗方案等基线资料作为处理变量，按照1∶1进行了倾向性匹配分析，匹配后两组各纳入56例患者，两组患者基线特征均一致（*P*>0.1）。

二、LT3S在AML患者中的预后意义

1. 生存分析：中位随访时间37.8（23.3～50.3月）个月。Kaplan-Meier分析显示，LT3S组的中位OS和PFS时间明显短于非LT3S组（OS：7.5个月对29.9个月，*P*<0.001；PFS：2.0个月对24.0月，*P*＝0.000）（图1A、B）。并且，合并LT3S的AML患者早期死亡率（20/62，32.3％）明显高于未合并LT3S者（21/174，12.1％）（*P*＝0.000）。使用倾向性匹配评分均衡患者基线资料后显示，LT3S组与非LT3S组相比OS时间（9.6个月对30.4个月，*P*＝0.010）和PFS时间（3.0个月对30.0个月，*P*＝0.014）仍明显缩短（图1C、D），但两组的早期死亡率差异无统计学意义（28.8％对17.3％，*P*＝0.163）。

**图1:**
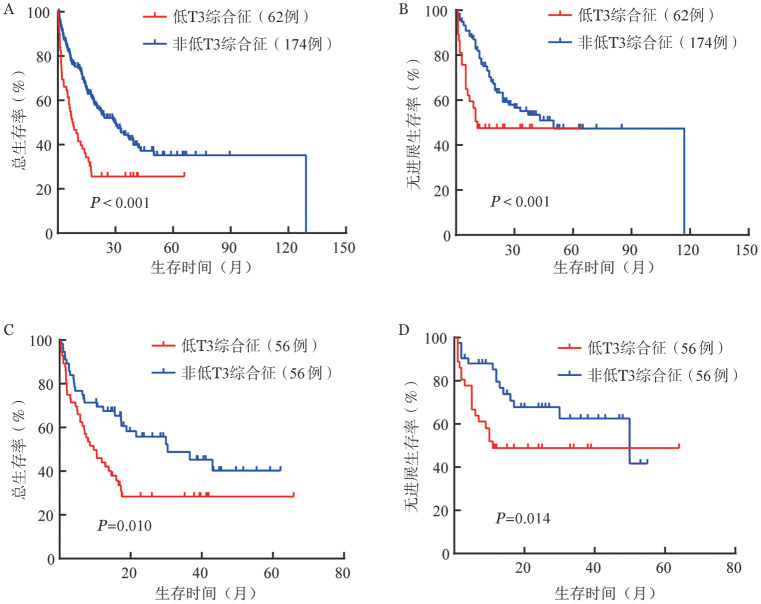
低T3综合征组和非低T3综合征组急性髓系白血病患者生存分析 A、B分别为倾向性匹配评分前总生存和无进展生存分析；C、D分别为倾向性匹配评分后总生存和无进展生存分析

此外，我们发现FT3和FT4同步减低的患者中位OS时间（5.8个月对10.4个月，*P*＝0.005）和PFS时间（1.0个月对5.0个月，*P*＝0.004）比单纯FT3减低组患者更短（图2A、B）。我们还对19例合并LT3S的患者进行化疗前后甲状腺功能的比较，结果发现，化疗缓解后，血清FT3水平明显恢复（*P*＝0.038），而FT4和TSH水平在治疗前后无显著变化（图3）。

**图2:**
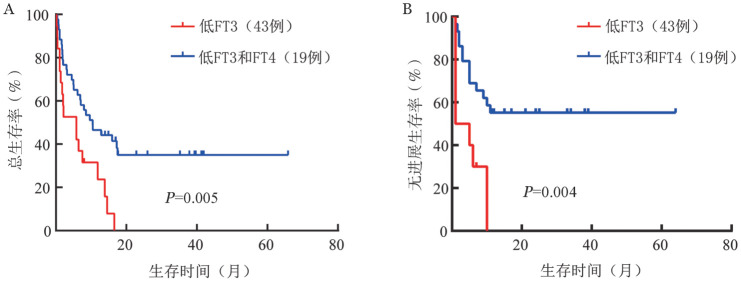
低游离三碘甲状腺原氨酸（FT3）和游离甲状腺素（FT4）对急性髓系白血病患者总生存（A）和无进展生存（B）的影响

**图3:**
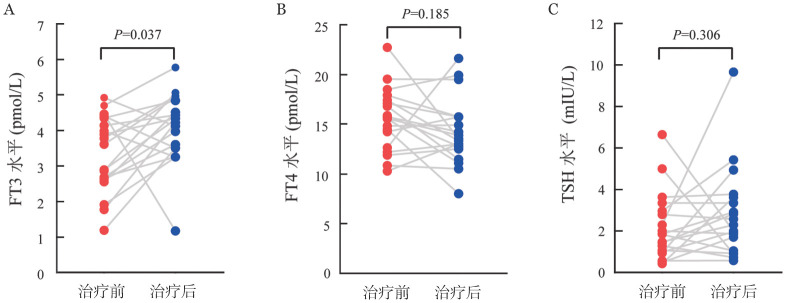
急性髓系白血病患者治疗前后血清FT3（A）、FT4（B）、TSH（C）水平变化 FT3：游离三碘甲状腺原氨酸；FT4：游离甲状腺素；TSH：促甲状腺激素

2. Cox回归分析：为了证实LT3S在AML患者中的预后意义，我们对可能影响AML预后的潜在风险预测指标进行了单因素和多因素Cox回归分析，结果见表1。多因素分析显示，年龄≥60岁、HGB<90 g/L、ECOG评分≥3、LT3S、TP53突变或缺失、欧洲白血病网（ELN）预后分型是影响AML患者OS和PFS的独立预后因素。其中LT3S与较差的OS（*HR*＝2.553，95％ *CI* 1.666～3.912，*P*<0.001）和PFS（*HR*＝1.701，95％ *CI* 1.114～2.597，*P*＝0.014）之间存在相关性。倾向性匹配评分后，多因素分析显示，LT3S仍为影响AML患者OS的独立预后不良因素（*HR*＝2.200，95％*CI* 1.287～3.761，*P*＝0.004）。对于PFS，合并LT3S的AML患者PFS低于非LT3S患者，但两者之间差异无统计学意义（*HR*＝1.684，95％*CI* 0.991～2.740，*P*＝0.054）（表2）。

**表1:** 倾向性匹配评分前影响急性髓系白血病患者OS和PFS的单因素和多因素分析

因素	单因素分析				多因素分析			
	OS		PFS		OS		PFS	
	*HR* (95% *CI*)	*P*值	*HR* (95% *CI*)	*P*值	*HR* (95% *CI*)	*P*值	*HR* (95% *CI*)	*P*值
低T3综合征	2.094(1.451~3.021)	<0.001	1.857(1.285~2.683)	0.001	2.553(1.666~3.912)	<0.001	1.701(1.114~2.597)	0.014
年龄≥60岁	4.104(2.808~5.997)	<0.001	3.353(2.302~4.883)	<0.001	3.484(2.211~5.491)	<0.001	2.691(1.727~4.195)	<0.001
男性	1.054(0.751~1.480)	0.759	1.063(0.757~1.492)	0.725	−	−	−	−
WBC≥20×10^9^/L	0.925(0.648~1.319)	0.666	0.944(0.659~1.353)	0.755	−	−	−	−
HGB<90 g/L	1.839(1.263~2.678)	0.001	1.810(1.243~2.637)	0.002	1.902(1.221~2.963)	0.004	1.738(1.136~2.660)	0.011
PLT<100×10^9^/L	0.872(0.582~1.305)	0.505	0.838(0.560~1.254)	0.390	−	−	−	−
LDH>271 U/L	1.238(0.879~1.743)	0.222	1.126(0.799~1.586)	0.498	−	−	−	−
ALB<30 g/L	1.708(1.086~2.686)	0.020	1.409(0.897~2.212)	0.137	−	−	−	−
NPM1突变	0.926(0.593~1.445)	0.734	0.939(0.602~1.466)	0.783	−	−	−	−
FLT3突变	1.039(0.651~1.656)	0.873	1.099(0.689~1.752)	0.692	−	−	−	−
TP53突变	2.723(1.718~4.315)	<0.001	2.256(1.420~3.584)	0.001	2.494(1.549~4.015)	<0.001	1.652(1.019~2.677)	0.042
ECOG评分 ≥3	3.544(2.246~5.592)	<0.001	2.719(1.718~4.303)	<0.001	4.117(2.435~7.166)	<0.001	2.309(1.367~3.899)	0.002
BMI>24 kg/m^2^	0.941(0.671~1.319)	0.724	0.920(0.654~1.296)	0.635	−	−	−	−
ELN预后分型								
良好	参考组				参考组			
中等	2.006(1.315~3.058)	0.001	1.991(1.308~3.031)	0.001	2.101(1.320~3.344)	0.002	1.920(1.214~3.038)	0.005
不良	5.004(3.314~7.554)	<0.001	3.723(2.454~5.649)	<0.001	4.582(2.870~7.317)	<0.001	2.623(1.633~4.214)	<0.001

注: T3：三碘甲状腺原氨酸；OS：总生存；PFS：无进展生存；LDH：乳酸脱氢酶；ALB：白蛋白；ECOG：美国东部肿瘤协作组；BMI：体重指数；ELN：欧洲白血病网；−：单因素分析不满足*P*<0.1的变量未纳入多因素分析

**表2:** 倾向性匹配评分后影响急性髓系白血病患者OS和PFS的单因素和多因素分析

因素	单因素分析				多因素分析			
	OS		PFS		OS		PFS	
	*HR* (95% *CI*)	*P*值	*HR* (95% *CI*)	*P*值	*HR* (95% *CI*)	*P*值	*HR* (95% *CI*)	*P*值
低T3综合征	1.915(1.162～3.158)	0.011	1.761(1.070～2.899)	0.026	2.200(1.287～3.761)	0.004	1.684(0.991～2.740)	0.054
年龄≥60岁	4.873(2.609～9.100)	<0.001	3.903(2.096～7.266)	<0.001	3.790(1.986～7.235)	<0.001	3.330(1.759～6.306)	<0.001
男性	1.075(0.656～1.762)	0.774	1.065(0.650～1.746)	0.801	−	−	−	−
WBC≥20×10^9^/L	0.782(0.465～1.316)	0.355	0.789(0.469～1.328)	0.372	−	−	−	−
HGB<90 g/L	1.682(0.956～2.959)	0.071	1.686(0.957～2.968)	0.071	1.833(0.977～3.441)	0.059	1.598(0.878～2.908)	0.125
PLT<100×10^9^/L	0.965(0.515～1.808)	0.911	0.813(0.434～1.524)	0.519	−	−	−	−
LDH>271 U/L	0.840(0.508～1.389)	0.498	0.919(0.554～1.526)	0.745	−	−	−	−
ALB<30 g/L	1.467(0.824～2.614)	0.193	1.323(0.742～2.359)	0.342	−	−	−	−
NPM1突变	0.842(0.458～1.546)	0.579	0.828(0.450～1.521)	0.543	−	−	−	−
FLT3突变	0.969(0.494～1.903)	0.928	1.000(0.509～1.964)	1.000	−	−	−	−
TP53突变	2.989(1.529～5.842)	0.001	2.280(1.171～4.439)	0.015	1.904(0.942～3.848)	0.073	1.281(0.625～2.627)	0.499
ECOG评分≥3	6.250(2.946～13.262)	<0.001	2.985(1.402～6.354)	0.005	7.522(3.101～18.244)	<0.001	2.331(1.049～5.178)	0.038
BMI >24 kg/m^2^	1.181(0.725～1.924)	0.503	1.124(0.691～1.829)	0.638	−	−	−	−
ELN预后分型								
良好	参考组							
中等	1.489(0.805～2.754)	0.204	1.415(0.765～2.619)	0.269	−	−	−	−
不良	1.632(0.803～3.317)	0.176	1.423(0.693～2.920)	0.337	−	−	−	−

注：T3：三碘甲腺原氨酸；OS：总生存；PFS：无进展生存；LDH：乳酸脱氢酶；ALB：白蛋白；ECOG：美国东部肿瘤协作组；BMI：体重指数；ELN：欧洲白血病网；−：单因素分析不满足*P*<0.1的变量未纳入多因素分析

3. 敏感性分析：为了避免混杂因素的干扰，保证结果的可靠性，我们进行了敏感性分析，分别将年龄>75岁、ECOG评分>3、采用姑息治疗的患者剔除，再次进行生存分析。结果显示在排除上述高龄、体能状态差、姑息治疗等离散值后，LT3S组的OS时间（年龄≤75岁：10.5个月对32.7个月，*P*<0.001；ECOG评分≤3：9.6个月对29.9个月，*P*<0.001；非姑息治疗：10.4个月对29.9个月，*P*<0.001）和PFS时间（年龄≤75岁：5.0个月对24.0个月，*P*＝0.001；ECOG评分≤3：3.0个月对21.0个月，*P*＝0.001；非姑息治疗：5.0个月对21.0个月，*P*＝0.002）仍显著低于非LT3S组的患者。

4. 亚组分析：进一步我们还进行了亚组分析，以了解LT3S在不同亚组患者中的预后意义，包括年龄、性别、HGB、TP53突变、ECOG评分、BMI、ALB和LDH。交互作用检验结果显示，对于OS，仅按照治疗方案（交互检验：*P*＝0.001）、ECOG评分（交互检验：*P*＝0.006）和BMI（交互检验：*P*＝0.029）进行亚组分析有统计学意义，提示LT3S的预后意义在不同的年龄、性别、TP53突变状态以及HGB、ALB、LDH水平的AML患者中无明显差异，而在采用标准方案化疗、ECOG评分≥3、BMI>24 kg/m^2^的AML患者中，合并LT3S者预后更差（表3）。对于PFS，如表3所示，在采用标准方案化疗和BMI>24 kg/m^2^的AML患者中，合并LT3S者PFS时间更短，预后更差。

**表3:** 低T3综合征与急性髓系白血病患者临床参数交互作用分析

临床特征	OS			PFS		
	*HR（95%CI）*	*P*值	交互作用	*HR（95%CI*）	*P*值	交互作用
年龄(岁)						
<60	1.92（0.97～3.81）	0.062	0.871	1.91（0.96～3.80）	0.064	0.425
≥60	1.83（1.19～2.80）	0.006		1.46（0.95～2.26）	0.084	
性别						
男	1.83（1.12～3.00）	0.016	0.377	1.66（1.01～2.72）	0.053	0.497
女	2.53（1.47～4.36）	0.001		2.18（1.27～3.75）	0.005	
治疗方案						
DCAG	1.31（0.78～2.20）	0.306	0.001	1.22（0.73～2.04）	0.451	0.002
标准治疗	2.83（1.57～5.12）	0.001		2.71（1.50～4.90）	0.001	
HGB（g/L）						
<90	2.47（1.21～5.02）	0.013	0.486	2.05（1.01～4.16）	0.047	0.571
≥90	1.75（1.15～2.68）	0.010		1.59（1.04～2.44）	0.033	
TP53						
突变型	2.40（0.96～6.01）	0.061	0.645	1.50（0.60～3.72）	0.387	0.663
野生型	2.094（1.41～3.12）	<0.001		1.92（1.28～2.86）	0.001	
ECOG评分						
0～2	2.02（1.35～3.01）	0.001	0.006	1.86（1.24～2.77）	0.002	0.996
≥3	6.01（2.14～16.89）	0.001		2.51（0.92～6.85）	0.073	
BMI（kg/m^2^）						
≤24	1.52（0.93～2.51）	0.097	0.029	1.38（0.84～2.27）	0.211	0.036
>24	3.34（1.91～5.82）	<0.001		2.80（1.60～4.90）	<0.001	
ALB（g/L）						
<30	1.70（0.75～3.90）	0.205	0.892	1.36（0.59～3.11）	0.467	0.762
≥30	2.06（1.36～3.12）	0.001		1.86（1.23～2.82）	0.003	
LDH（U/L）						
≤271	1.88（1.08～3.27）	0.026	0.658	1.59（0.92～2.78）	0.100	0.620
>271	2.41（1.47～3.93）	<0.001		2.20（1.34～3.62）	0.002	

注：OS：总生存；PFS：无进展生存；LDH：乳酸脱氢酶；ALB：白蛋白；BMI：体重指数；ECOG：美国东部肿瘤协作组；DCAG：地西他滨+阿糖胞苷+阿柔比星+G-CSF

## 讨论

LT3S可在很多临床状态下发生，如饥饿、创伤、感染、肿瘤、心脏及肾脏疾病等。目前，关于LT3S的发生机制仍缺乏定论，部分观点认为T3在机体内主要参与代谢，血清T3水平下降是机体在应激状态下减慢代谢、降低消耗的保护性机制[9]。然而也有研究者认为，LT3S，尤其是伴低T4者，可能是危重疾病或应激损伤造成的结果而并非原因，其与疾病的进展和预后不良有潜在联系[10]。前期研究表明，在慢性淋巴细胞白血病和噬血细胞综合征患者中，LT3S的发生率分别为13.34％和75.7％，并且与预后不良有关[11]–[12]。然而，目前尚无关于LT3S在AML治疗中临床意义的研究，因此，在本研究中，我们首次评估了LT3S对AML患者OS、PFS和早期死亡率的影响。

在236例AML患者中，26.3％的患者在初诊时合并LT3S，并且血清FT3水平与血清ALB、HGB、CRP、LDH水平呈明显的相关性。表明LT3S在AML患者中发生率很高，并且FT3水平可能与蛋白代谢、炎症、肿瘤负荷等指标相关。这与Fan等[13]在慢性肾脏病中的研究结果一致。研究认为LT3S的发生与应激状态下肝脏产生甲状腺激素结合球蛋白功能受抑，白蛋白水平下降有关[14]。并且已证实白细胞介素-6（IL-6）、肿瘤坏死因子（TNF）-α等炎性细胞因子均参与了LT3S的发生[15]，而在AML中，髓系原始细胞可诱导产生一系列炎症介质，包括脂质、趋化因子、细胞因子和生长因子等[16]。因此，LT3S的发生表明机体处于一种蛋白代谢异常、炎症应激的状态。

研究结果表明，在合并LT3S的AML患者中，中位OS及PFS时间显著短于未合并LT3S的患者，并且早期死亡率高达32.3％，这提示我们LT3S是影响预后、尤其是早期死亡的重要因素。此外，通过比较治疗前后血清T3的水平，发现治疗缓解后血清T3水平明显上升，进一步说明LT3S可以作为一个反映临床状态的指标。急性白血病侵袭性高，LT3S的发生反映患者处于疾病的严重或应激状态，而这种临床状态下患者往往病死率高，且不能耐受高强度的白血病治疗，因此，早期识别LT3S可以帮助我们在治疗前和治疗过程中进行评估，制定个体化治疗方案。

由于我们的研究是回顾性分析，为了避免混杂因素的影响，我们进行了倾向性匹配评分，在基线特征均衡的112例患者中（56对56）进一步证明了LT3S是影响AML患者预后的独立危险因素。此外，为了探讨LT3S在不同患者群体中的预后意义，我们还进行了亚组分析。结果表明，LT3S在AML患者中的预后意义，在不同的年龄、性别、是否伴有TP53突变以及不同HGB、ALB、LDH水平患者中均无明显差异。而在体能状态差（ECOG评分≥3）、肥胖（BMI>24 kg/m^2^）和采用标准方案化疗的AML患者中，合并LT3S可能具有更差的预后。研究表明慢性低度炎症是肥胖的一个特征[17]，这种炎症主要由脂肪组织释放的物质驱动，介导TNF-α、IL-1、IL-6等多种促炎细胞因子的产生[18]，从而可能参与LT3S的发生。此外，甲状腺激素是骨骼肌收缩、代谢、肌肉生成和再生所必需的内分泌激素[19]–[20]，LT3S患者可能伴随肌肉的消耗及损失。而已有明确的证据表明，肌肉损失、体能状态差的AML患者通常无法耐受强化治疗，肌少症与临床预后差具有相关性[21]。我们既往研究结果显示，标准方案化疗与DCAG方案相比，发生感染、心功能不全、转氨酶升高等不良反应的比例更高，患者耐受性更差[22]，因此，亚组分析的结果提示在合并LT3S的AML患者中，可能采用DCAG方案治疗更优。该结论需要后续临床研究进一步证实。综合以上结果表明，合并LT3S的肥胖和体能状态差组患者预后更差，可能不适合高强度的化疗方案，采用DCAG方案可能是适合该类患者的治疗方案。

对于合并LT3S的患者是否需要采用甲状腺激素替代治疗极具争议 [23]–[24]。后续我们将进一步开展前瞻性研究，探究在伴有LT3S的AML患者中采用甲状腺激素替代治疗能否改善患者的全身状态，提高AML患者的化疗效果。

综上所述，我们在研究中首次发现合并LT3S在AML患者中具有独立预后不良意义，甲状腺激素作为一种简单、易测量的参数，在评估AML患者的临床状态、风险分层和制定临床决策中具有重要意义。本研究为单中心、回顾性研究，未来我们将扩大样本量、延长随访时间，进一步明确LT3S在AML患者中的预测价值，并深入探讨LT3S影响AML患者预后的潜在机制。
